# Phenolic Profiling of Flax Highlights Contrasting Patterns in Winter and Spring Varieties

**DOI:** 10.3390/molecules24234303

**Published:** 2019-11-26

**Authors:** Job Tchoumtchoua, David Mathiron, Nicole Pontarin, David Gagneul, Anne-Isaline van Bohemen, Elvis Otogo N’nang, François Mesnard, Emmanuel Petit, Jean-Xavier Fontaine, Roland Molinié, Anthony Quéro

**Affiliations:** 1 EA 3900-BIOPI Biologie des Plantes et Innovation, Université de Picardie Jules Verne, Faculté de Pharmacie, 1 rue des Louvels, 80025 Amiens Cedex, France; tchoumtchoua@gmail.com (J.T.); nicole.pontarin@gmail.com (N.P.); ai.vanbohemen@gmail.com (A.-I.v.B.); elvis.oto@u-picardie.fr (E.O.N.); francois.mesnard@u-picardie.fr (F.M.); emmanuel.petit@u-picardie.fr (E.P.); jean-xavier.fontaine@u-picardie.fr (J.-X.F.); roland.molinie@u-picardie.fr (R.M.); 2 Plate-Forme Analytique, Université de Picardie Jules Verne, 33 rue Saint Leu, 80039 Amiens, France; david.mathiron@u-picardie.fr; 3 EA 7394, USC INRA 1411, Institut Charles Viollette (ICV), Agro-food and Biotechnology Research Institute, Université de Lille, INRA, ISA, Univ. Artois, Univ. Littoral Côte d’Opale, Cité Scientifique, 59655 Villeneuve d’Ascq, France; david.gagneul@univ-lille.fr

**Keywords:** flax, *C*-glycosyl-flavonoids, UPLC-MS, NMR, cold tolerance

## Abstract

Flax (*Linum usitatissimum*) is a plant grown in temperate regions either for its fiber or for its seeds, which are rich in the essential fatty acid omega-3. It is also well known as a source of medicinal compounds. The chemical composition of its leaves is currently poorly described. In order to fill this gap, we have conducted a comprehensive analysis of flax leaf metabolome. The exploration of the metabolome allowed the characterization of compounds isolated for the first time in flax leaves. These molecules were isolated by preparative HPLC and then characterized by NMR, LC-MS and standard analysis. This work extended our picture of *C*-glycosyl-flavonoids and coniferyl alcohol derivatives accumulated in flax. The follow-up of the content of these different metabolites via UPLC-MS revealed significant accumulation differences in spring and winter flax leaves. In particular, two methylated *C*-glycosylflavonoids (swertisin and swertiajaponin) were the most abundant phenolic compounds in winter flax whereas they were not detected in spring flax. This result suggests that these 2 compounds are involved in cold stress tolerance in flax.

## 1. Introduction

Plants accumulate a wide array of molecules classified into two categories: primary metabolites and specialized metabolites. The latter are also known as secondary metabolites because unlike primary metabolites, they are not directly responsible for the growth and development of plants under standard growth conditions. These compounds represent active ingredients in several medicinal plants. The appearance of these molecules during plant evolution has largely contributed to the tremendous adaptability of plants to their changing environment. They are involved in ecological interactions such as pollinator attraction, herbivore defense or abiotic stress protection [1]. During plant evolution, distinct metabolic pathways arise from gene neofunctionalization following gene duplication. Depending on the evolutionary advantage conferred by the newly synthesized molecules, the pathways are then maintained or not [2]. This explains why specialized metabolites are often lineage specific compounds.

Flavonoids, a class of phenolics, are well known for their biological properties and are associated with a range of human health-related benefits [3]. In plants, these phytochemicals serve essential functions in plant reproduction, namely pollination and seed dispersal [4]. In addition, they are involved in a wide range of important physiological activities and play an important role in plant protection against various abiotic and biotic stresses [5]. Their 15-carbon basic skeleton is synthesized from the combination of coumaroyl-CoA arising from the phenylpropanoid pathway and malonyl-CoA, which is an intermediate of the fatty acid metabolism. The derived chalcone is further modified by oxygenases, methyltransferases, and glycosyltransferases to give a wide diversity of flavonoids belonging to various classes i.e., flavanones, isoflavones, flavanols, anthocyanins, and flavones. More than 8000 structurally different flavonoids are produced and accumulated by plants [6]. Glycosylation of flavonoids is an important feature that modifies their stability, solubility, subcellular localization and biological properties. Two types of glycosylation can occur [7]. The *O*-glycosylation involves a linkage between the glycosyl moiety and one of the hydroxyl group of the flavone backbone. In *C*-glycosylation, linkage occurs directly between the glycosyl moiety and one of the carbon of the genin. This latter glycosylation is more stable and *C*-glycosyl flavonoids are of particular interest for human health applications since they are expected to be more stable to degradation and to reach more efficiently their molecular target.

In plants, flavonoids are often associated with cold tolerance. The presence of low temperatures determines the geographical distribution of plants and limits the productivity of cultivated plants [8,9]. The cold stress tolerance differs depending on the intensity of the stress. Different degrees of tolerance can be shown toward chilling (when the temperatures are between 0 and 15 °C) and freezing (when the temperatures are negative) [10]. To tolerate these low temperatures, plants develop adaptive traits and modify the expression of their genes to adjust their metabolism. Under cold stress conditions, it has been shown that phenolic compounds, including flavonoids, accumulate in many plants, suggesting that they may be involved in cold stress tolerance [11,12,13].

Flax (*Linum usitatissimum* L.) is a dicotyledonous plant cultivated as a source of fiber, oil and medicinal compounds. Little information is known about the identity of phenolic compounds accumulated by flax and about their possible involvement in cold tolerance. This plant cultivated in temperate climates for its omega-3-rich oil exhibits contrasted tolerance to cold stress depending on the variety. Indeed spring cultivars are cold sensitive whereas winter varieties are considered as cold tolerant. 

Here, we report a comprehensive profiling of the main phenolic compounds accumulated in leaves of spring and winter flax. Combination of LC-MS and NMR enabled structure assignment to 18 compounds. In particular, 5 *C*-glycosylated flavonoids were identified for the first time in flax leaves. Interestingly, two of these compounds, swertiajaponin and swertisin, were shown to be specifically accumulated in winter varieties. These compounds may potentially have a role in cold stress tolerance in flax and may constitute target to direct future breeding strategies.

## 2. Results

### 2.1. Identification of Phenolic Compounds in Flax Leaves

**Figure 1:**
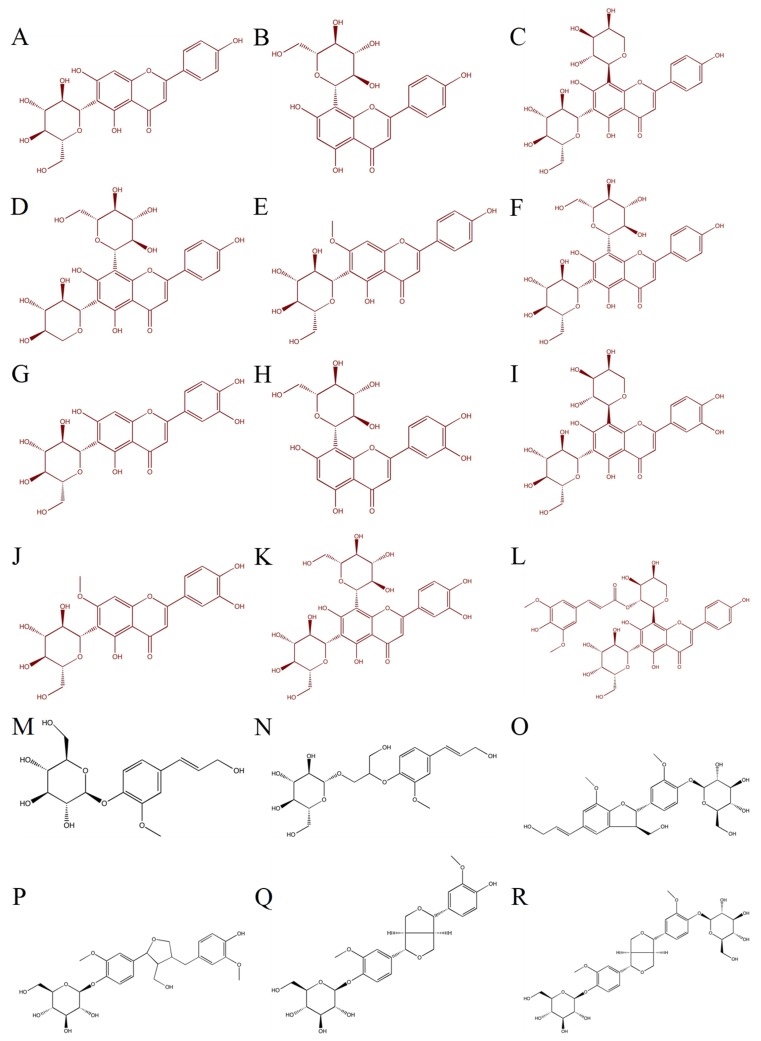
Structures of compounds profiled in flax leaves by LC-MS. (**A**) Isovitexin, (**B**) Vitexin, (**C**) Schaftoside, (**D**) Vicenin-1, (**E**) Swertisin, (**F**) Vicenin-2, (**G**) Isoorientin, (**H**) Orientin, (I) Carlinoside, (**J**) Swertiajaponin, (K) Lucenin-2, (**L**) Triticuside A, (**M**) Coniferin, (**N**) 3-Hydroxy-2-[4-[(1*E*)-3-hydroxy-1-propen-1-yl]-2-methoxyphenoxy]propyl β-d-glucoside (HHMPG), (**O**) Dehydro- diconiferyl alcohol-4-*O*-glucoside (DCG), (**P**) Lariciresinol-4-*O*-glucoside (LMG), (**Q**) Pinoresinol-4-*O*-glucoside (PMG), (**R**) Pinoresinol diglucoside (PDG).

The phytochemical composition of flax leaves is not well documented. We carried out an extensive analysis of compounds present in a hydroalcoholic leaf extract of winter flax. To assign a structure to these compounds, UV major peaks were collected and analyzed by LC-MS-MS and features (retention time, fragmentation) compared to commercial standards of compounds already described in flax leaves [14]. As a result, the identities of 7 *C*-glycosylflavonoids were confirmed: vitexin, isovitexin, orientin, isoorientin, vicenin-1, vicenin-2 and lucenin-2 (Figure 1). Among the collected peaks, 4 remaining fractions did not match to any compounds previously identified in flax. LC-MS and NMR experiments were then carried out in order to identify these compounds.

#### 2.1.1. Swertisin Characterization

Compound E displayed the molecular formula C_22_H_22_O_10_ on the basis of accurate mass measurement of its deprotonated molecule [M − H]^−^ obtained by ESI-HRMS (*m/z* measured 445.1155; *m/z* calculated 445.1140; error (ppm) 3.4). ^1^H- and ^13^C-NMR data are presented in Supplementary Materials 1. The ^1^H-NMR spectrum exhibited a flavonoid pattern with four aromatic signals: an AA′BB′ system at δ_H2’/H6’_ 7.91 (2H, d, *J* = 8.8 Hz) and δ_H3’/H5’_ 6.95 (2H, d, *J* = 8.8 Hz), and two additional aromatic signals at δ_H3_ 6.69 (1H, s) and δ_H8_ 6.77 (1H, s). The carbon atoms of the aforementioned protons were found to resonate at 128.0 ppm (C-2′/C-6′), 115.8 ppm (C-3′/C-5′), 102.9 ppm (C-3) and 89.9 ppm (C-8), respectively. The methoxy protons were observed as a singlet at δ 3.96 and the corresponding carbon atom at 55.3 ppm. The HMBC spectrum revealed a correlation of the hydroxyl group with C-4′ (161.6 ppm) and the methoxy with C-7 (165.1 ppm) confirming their position. The ^1^H-NMR shifts at δ 3.39–4.23 and signals in the HSQC spectrum 61.9–81.2 ppm indicated the presence of a glucose moiety. The doublet signal at δ_H1”_ 4.90 (1H, *J* = 10.3 Hz) with the corresponding carbon atom at C-1” (73.0 ppm) was assigned to the anomeric proton and indicated a *C*-β-configuration. Hence, compound E was assigned as swertisin and confirmed by the data of previous literature [15]. This characterization has been confirmed by comparison to swertisin commercial standard which presented the same retention time, the same exact mass and the same MS/MS fragmentation pattern than the compound E (Supplementary Materials 2).

#### 2.1.2. Swertiajaponin Characterization

Compound J displayed the molecular formula C*_22_*H_22_O_11_ as determined by accurate mass measurement by ESI-HRMS of its deprotonated molecule [M − H]^−^ (*m/z* measured 461.1107; *m/z* calculated 461.1089; error (ppm) 3.9). According to ^1^H- and ^13^C-NMR data (Supplementary Materials 3), compound J was structurally close to swertisin described above. The aromatic signals at δ_H3_ 6.62 (1H, s) and δ_H8_ 6.74 (1H, s) with their corresponding carbons resonating at 102.6 ppm (C-3) and 89.5 ppm (C-8) were identified. The methoxyl protons were observed as a singlet at δ 3.95 with the corresponding carbon atom at 55.0 ppm and HMBC correlation to C-7 (165.0 ppm). The anomeric signal of the *C*-β-glucoside moitie resonated as a doublet at δ_H-1′′_ 4.89 (1H, *J* = 10.0 Hz) with the corresponding carbon atom at 72.7 ppm. The main difference was observed at B-ring where an ABX system consisting of a doublet of doublets at δ_H6’_ 7.44 (1H, dd, *J* = 2.1/8.4 Hz) and two doublets δ_H2’_ 7.41 (*J* = 2.1 Hz) and δ_H5’_ 6.92 (*J* = 8.4 Hz) was noticed. This observation is in agreement with the meta and para substitution on B ring by hydroxyl groups. The carbon atoms of these protons were found to resonate at 112.6 ppm (C-2′), 115.0 ppm (C-5′) and 118.6 ppm (C-6′), respectively. Hence, compound J was assigned as swertiajaponin and confirmed by the data of previous literature [15]. This characterization has been confirmed by comparison to swertiajaponin commercial standard which presented the same retention time, the same exact mass and the same MS/MS fragmentation pattern than the compound J (Supplementary Materials 2).

#### 2.1.3. Triticuside A Characterization

In the purified fraction, two chromatographic peaks with a ratio 1:5were detected by LC-ESI-HRMS at R_t_ 4.82 and 4.91 min, respectively. The comparison of corresponding ESI-HRMS spectra highlighted the presence of isobaric ions whose accurate mass measurement of their deprotonated molecules [M − H]^−^ (*m/z* measured 769.1983; *m/z* calculated 769.1985; error (ppm) −0.3) resulted in the predicted molecular formula C_37_H_38_O_18_ in both cases. Because of their close retention times and of their identical molecular formula, these two compounds were considered as isomers. Thereafter, the structural characterization of the main isomer by NMR (Compound L) was conducted.

^1^H- and ^13^C-NMR data are presented in Supplementary Materials 4. The ^1^H-NMR spectrum exhibiting four aromatic signals: an AA′BB′ system at δ_H2’/H6’_ 8.07 ppm (2H, d, *J* = 8.9 Hz) and δ_H3’/H5’_ 6.98 ppm (2H, d, *J* = 8.9 Hz), and an additional aromatic signals at δ_H3_ 6.67 ppm (1H, s) is consistent with a flavonoid pattern. HMBC correlations H2’-H6’/C2, H3/C2 and the low intensity cross peak H3/C4 confirmed our previous ^1^H assignments and on the other hand revealed the linkage between B-ring and C-ring on the position 2. Other HMBC informative correlations were observed on the B-ring between H2’–H6’ and C4’ and on the C-ring between H3 and C10. The absence of aromatic ^1^H signal detected on A-ring implies the total substitution of this cycle. Elsewhere in the ^1^H spectrum, 7’’–8’’ unsaturation was found as two doublets at δ_H7’’_ 7.47 ppm (1H, d, *J* = 15.9 Hz) and δ_H8’’_ 6.21 ppm (1H, d, *J* = 15.9 Hz) which coupling constant value corresponds to a trans-stereochemistry. Moreover, a singlet corresponding to an aromatic signal at δ_H2’’-H6’’_ 6.87 ppm (2H, s) and one singlet resonating at 3.87 ppm assigned to protons from methoxy groups were observed. As previously, ^1^H-^13^C HMBC experiment was helpful to complete the structural elucidation. Some correlations were highlighted in the aromatic cycle such as OMe protons with C3” and C5” and H2”–H6”/C4”. Then, a correlation H2”–H6”/C7” clarified the link between the aromatic system and the double bond. The link between the double bond and the ester carbonyl was evidenced by the correlation H7”/C9”. As a partial conclusion, the presence of a sinapoyl unit can be assumed. Finally, characteristic ^1^H- and ^13^C-NMR sugar signals have been assigned to arabinosyl and galactosyl moieties thanks to 1D- and 2D-NMR experiments. The glycosidic linkage of the arabinosyl unit on the flavonoid A-ring has been confirmed by HMBC correlations occurring between H1’’’’/C7 and H1’’’’/C9. The grafting of the galactosyl unit on the flavonoid A-ring has been revealed by the presence of HMBC correlations between H1’’’/C5 and H1’’’/C7.

To summarize, all the structural investigations led by NMR have demonstrated the presence of a flavonoid *C*-glycoside structure with a grafted sinapoyl group. By comparing our NMR data with the literature [16], the ^1^H, ^13^C chemical shift values of our compound have been found to be closer to those of triticuside A than to those of triticuside B. As previously described in *Triticum aestivum* L [16], our NMR data suggested the presence of a mixture of two isomers (triticuside A and B) whose the major compound (L) seems to be apigenin-6-*C*-galactoside-8-*C*-(2-sinapoyl) arabinoside (triticuside A) in our purified fraction.

To confirm the grafting position of the sinapoyl moiety on the flavonoid *C*-glycoside, LC-ESI-MS/HRMS experiment was performed on the [M − H]^−^ ion at *m/z* 769.20 to gain deeper structural information (Supplementary Materials 4). For the major peak (R_t_ 4.91 min), from the precursor ion at *m/z* 769.1987, a loss of 224.0684 u (C_11_H_12_O_5_) corresponding to sinapic acid was observed to obtain the fragment ion at *m/z* 545.1303 which is the base peak. The second fragmentation involved a loss of 120.0423 u (C_4_H_8_O_4_) from the ion at *m/z* 545.1303 to give the ion at *m/z* 425.0880. This loss can only be explained by an intra-cyclic cleavage ^0,2^X in the hexose unit [17]. Thus, the fragment ion at *m/z* 425.0880 is diagnostic since it evidences the structure to be a *C*-glycosylated flavonoid and it can also be considered as an indirect evidence of the substitution of the sinapoyl in position 2”” of the pentose. Indeed, once the sinapic acid loss in position 2”” occurred, the double bond formed in the pentose unit prevents its ^0,2^X cross-ring cleavage and seems to favor the fragmentation towards the cross-ring cleavage ^0,2^X in the hexose unit. Conversely for the minor isomer (R_t_ 4.82 min), once the sinapic acid has been lost on the hexose unit to give the fragment ion at *m/z* 545.1304, the cross-ring cleavage ^0,2^X occurring in the hexose unit being impossible, the fragmentation was reoriented towards a ^0,2^X cleavage in the pentose unit (−90.0314 u) to give the ion at *m/z* 455.0990. This fragmentation behavior has already been described to distinguish triticuside A from triticuside B [17] and is in agreement with our MS/MS results. To conclude, in the purified fraction the major compound (L) corresponds to triticuside A.

#### 2.1.4. HHMPG Characterization

Compound N molecular formula as C_19_H_28_O_10_ was determined by means of ESI-HRMS with the accurate mass measurement of its deprotonated molecule [M − H]^−^ (*m/z* measured 415.1623, *m/z* calculated 415.1610; error (ppm) 3.1). The ^1^H-NMR spectrum of compound N exhibited aromatic, olefinic, aliphatic and sugar signals. Aromatic signals were found as one doublet of doublets at δ_H5’_ 6.94 ppm (1H, dd, *J* = 2.1 and 8.2 Hz) and two doublets at δ_H3’_ 7.06 ppm (1H, d, *J* = 2.1 Hz) and δ_H6’_ 7.07 ppm (1H, d, *J* = 8.2 Hz). A singlet resonating at δ_2’-OMe_ 3.86 ppm was assigned to protons from methoxy group. The latter revealed a HMBC correlation with C2’ evidencing the link between the methoxy group and the aromatic region in the 2’position. Olefinic signals were found as two doublets at δ_H7’’_ 6.56 ppm (1H, d, *J* = 15.9 Hz) and δ_H8’’_ 6.28 ppm (1H, d, *J* = 15.9 Hz) whose coupling constant value corresponds to a *trans*-stereochemistry. The aliphatic part of the compound can be suggested by the presence of three signals at δ_H1_ 3.80 ppm (2H, dd, *J* = 4.7 and 12.2 Hz), δ_H2_ 4.42 ppm (1H, m) and δ_H3_ 3.84 and 4.11 ppm (2H, m and q, *J* = 5.4 Hz) which have been assigned to a glycerol residue. Characteristic sugar signals have been observed at δ_H1’’_ 4.33 ppm (1H, d, *J* = 7.8 Hz), δ_H2’’_ 3.20 ppm (1H, dd, *J* = 7.8 and 9.8 Hz), δ_H3’’_ 3.35 ppm (1H, m), δ_H4’’_ 3.30 ppm (1H, m), δ_H5’’_ 3.29 ppm (1H, m) and δ_H6’’_ 3.66 and 3.87 ppm (2H, m). The linkage of the C3 unsaturated chain (C7’ to C9’) was highlighted by the presence of the HMBC correlation H8’/C4’ and by the correlations occurring between H3’-5’/C7’, H7’/C3’-5’. The methoxyphenoxy moiety was linked to the glycerol residue (C1 to C3) as evidence by the HMBC correlation between H2/C1’, while the correlations between H3/C1’’ and H1’’/C3 revealed the glycosidic linkage at C3 with the glucose moiety. With all the structural information collected thanks to HMBC experiment and by comparing our ^1^H- and ^13^C-NMR data (Supplementary Materials 5) with previous literature [18], compound N was assigned as 3-hydroxy-2-{4-[(1*E*)-3-hydroxyprop-1-en-1-yl]-2-methoxyphenoxy}propyl-β-d-glucopyranoside (HHMPG).

**Figure 2:**
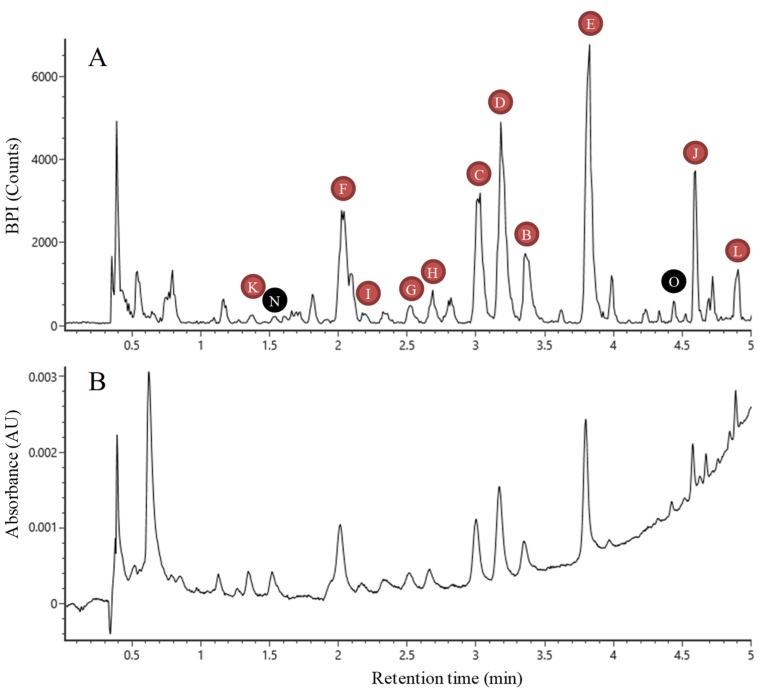
Chromatogramm of winter flax winter leaves extract obtained by LC-MS (**A**) and UV (**B**). B: Vitexin, C: Schaftoside, D: Vicenin-1, E: Swertisin, F: Vicenin-2, G: Isoorientin, H: Orientin, I: Carlinoside, J: Swertiajaponin, K: Lucenin-2, L: Triticuside A, N: HHMPG, O: DCG. The signals of coniferin, isovitexin, LMG, PMG and PDG were too low to be indicated on the chromatogramm.

From the aforementioned results, this work of characterization has expanded the vision of the phenolic compounds present in flax leaves. These compounds are mainly represented by *C*-glycosylflavonoids which are derived from 2 flavones aglycones (apigenin or luteolin). To complete this characterization work, commercial standards of other *C*-glycosylflavones derived from these two flavones aglycones were analyzed. This work helped identifying carlinoside and schaftoside from retention time, exact mass and MS/MS fragmentation obtained for the authentic standard. Similarly, the authentic standards of coniferin, lariciresinol-4-*O*-glucoside (LMG), pinoresinol-4-*O*-glucoside (PMG) and pinoresinol diglucoside (PDG) were analyzed to complete the picture of coniferyl alcohol derivatives. An authentic standard of DCG was also used to confirm its occurrence. This compound had been previously characterized in our laboratory in a previous study in *Linum usitatissimum* cell cultures [19]. Finally, 18 phenolic compounds were identified in flax leaves. All the characterized compounds are shown in Figure 1. They correspond to the major signals obtained by LC-MS and LC-UV (Figure 2).

### 2.2. Profiling of Phenolic Compounds in Flax Leaves

**Figure 3:**
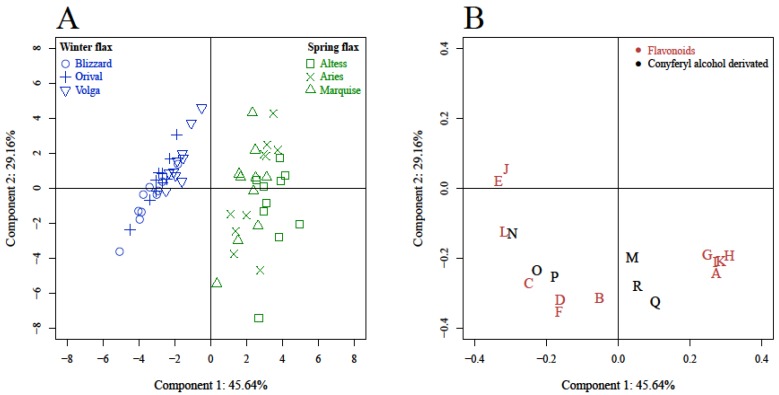
Principal component analysis (PCA) of flax leaves metabolites content with score plot (**A**) and loading plot (**B**). A: Isovitexin, B: Vitexin, C: Schaftoside, D: Vicenin-1, E: Swertisin, F: Vicenin-2, G: Isoorientin, H: Orientin, I: Carlinoside, J: Swertiajaponin, K: Lucenin-2, L: Triticuside A, M: Coniferin, N: 3-Hydroxy-2-[4-[(1*E*)-3-hydroxy-1-propen-1-yl]-2-methoxyphenoxy]propyl β-d-glucoside (HHMPG), O: Dehydrodiconiferyl alcohol-4-*O*-glucoside (DCG), P: Lariciresinol-4-*O*-glucoside (LMG), Q: Pinoresinol-4-*O*-glucoside (PMG), R: Pinoresinol diglucoside (PDG).

To identify compounds putatively involved in cold tolerance in flax, the relative contents of all identified phenolics were determined in the leaves of three spring varieties (Altess, Aries, Marquise) and three winter varieties (Blizzard, Orival, Volga). Principal component analysis (PCA) was then employed to examine the differences and similarities among the different varieties based upon the variation of their phenolic signature. The first two principal component axes cover 74.8% of the total sample variability with component 1 accounting for 45.6% and component 2 accounting for 29.2% of variability (Figure 3). Examination of the sample distribution shows clear discrimination along component 1 between samples obtained from spring varieties and those obtained from winter varieties. The projection of the variable metabolites in the plot defined by the two first axes is presented in Figure 3B. The positive part of the first principal component is strongly correlated with the highest values of a group of phenolics i.e., orientin, isoorientin, isovitexin, lucenin-2 and carlinoside. On the contrary, the negative part of principal component 1 is well correlated with swertisin, swertiajaponin, HHMPG and triticuside A. Accordingly, the leaves of winter varieties share a metabolic profile characterized by the highest contents of swertisin, swertiajaponin, HHMPG and triticuside A whereas the leaves of spring varieties are characterized by the highest values of orientin, isoorientin, isovitexin lucenin-2 and carlinoside.

**Figure 4:**
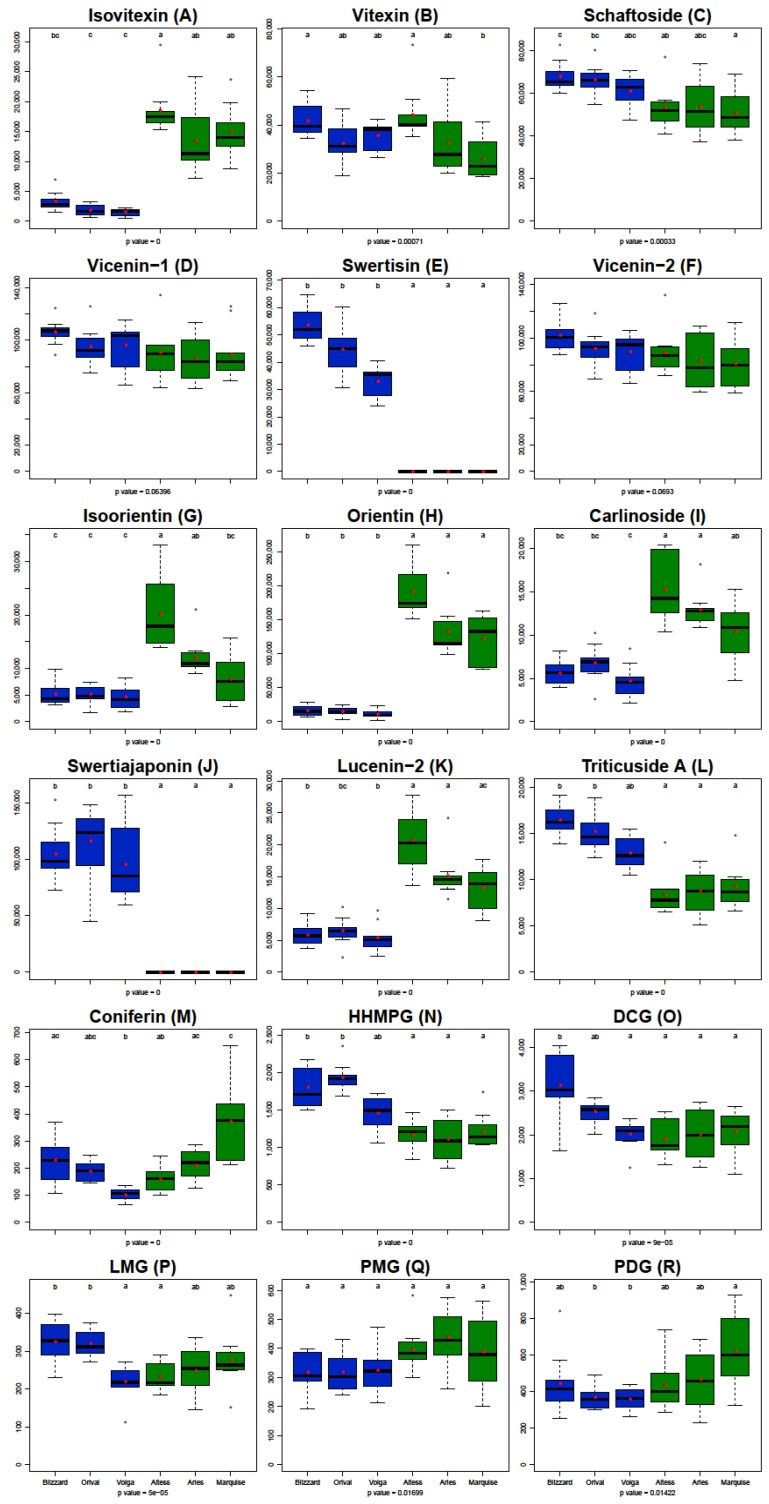
Boxplot of metabolite content in flax leaves. The results are expressed in area by mg of DW. Winter varieties are represented in blue and spring varieties are represented in green. Different letters indicate that the content were significantly different after the post-Hoc Kruskal-Wallis test (*p*-value < 0.05) for each compound. The red dots indicate the average of the variety.

Univariate analyses were performed using a Kruskal and Wallis test (*p* < 0.05) to monitor the relative differences in the content of each metabolite when comparing the winter and spring flax varieties (Figure 4). The main differences between the winter and spring flax varieties were obtained for the contents of swertisin and swertiajaponin. While the relative areas of these two compounds were among the highest in winter flax varieties, they were not detected (even as a trace) in spring flax varieties. In contrast, the contents of isovitexin (four to 12 times) and isoorientin, lucenin-2 and carlinoside (1.5 to four times) were significantly higher in spring flax varieties. Slight differences were detected for HHMPG, triticuside A and schaftoside. The contents of these three compounds in spring flax varieties were about 1.2 to two times lower than those in winter flax varieties.

Contents of other compounds did not discriminate spring and winter flax varieties. For coniferin, vitexin, DCG, PDG, LMG, differences were detected between varieties but they were not related to cold tolerance. The contents of PMG, vicenin-1 and vicenin-2 were identical in the different cultivars.

## 3. Discussion

The aim of this study was to carry a more thorough identification of phenolics accumulated in flax leaves and to investigate if their contents could differ between spring and winter flax varieties. Indeed flavonoid contents were shown to correlate with freezing tolerance in numerous plant species. Applications of several analytical techniques allowed the characterization of 18 main compounds. These compounds were essentially represented by *C*-glycosylflavones. These coumpounds are widespread in nature and they are the most abundant flavonoids in many plants such as rice, wheat and maize. Eight *C*-glycosyl flavones have already been described in flax leaves [14]. Five new *C*-glycosylflavones were characterized for the first time in flax: schaftoside, carlinoside, swertisin, swertiajaponin and triticuside A. All these compounds are structurally related to previously identified *C*-glycosyl flavones. Swertisin and swertiajaponin are structurally close to isovitexin and isoorientin, respectively, with an *O*-methyl group in the 7-position of the flavonoid backbone. Schaftoside and carlinoside are structurally close to isovitexin and isoorientin, respectively, with an arabinose in the 8-position of the flavonoid backbone. Triticuside A is derivated of apigenin and is conjugated with a hydroxycinnamic acid. These results suggest that all these compounds derive from common biosynthetic pathways and, at present, no *O*-glycosylated flavonoid was detected in flax leaves. In most plants, the main flavonoids are *O*-glycosylated derivatives but in some species (including most cereals) *C*-glycosyl flavonoids are the main compounds. This type of linkage is more resistant to hydrolysis than those of *O*-glycosylflavonoids. *C*-glycosylflavonoids are therefore more stable, which confers them a different biological activity from *O*-glycosylflavonoids [20]. In flax, the biological activities of these compounds are still poorly understood. Some studies suggest that these *C*-glycosylflavonoids are phytoalexins and that they may participate in the defense of the plant against pathogens because they exhibit antifungal activities against *Fusarium oxysporum* (necrotrophic pathogens of flax) [21]. The work presented here suggests that they could also be involved in cold stress tolerance. Winter and spring flax varieties can be easily distinguished based on their phenolic profiles. Notably swertisin and swertiajaponin were highly accumulated in winter flax varieties whereas they were not detected in spring flax varieties. This could suggest that these compounds have a role in cold stress protection. These two *C*-glycosylflavonoids are distinguished from other compounds by the presence of an *O*-methyl group in the 7-position of the flavonoid backbone. Although the biosynthetic pathway of these two compounds has not been extensively studied, experiments using radiolabeled precursors have shown that swertisin and swertiajponin are produced by *O*-methylation of either isovitexin or isoorientin, respectively [22]. The low presence of these 2 precursors in winter flax compared to spring flax could therefore be due to a remobilization of their pool for the synthesis of the methylated *C*-glycosylflavonoids. In plants the methylation of flavonoids is catalyzed by *O*-methyltransferases (OMT). The presence of *O*-methylated flavonoids in winter flax but not in spring flax suggests that ability to catalyze the methylation of isovitexin and isoorientin has been favored during the selection process. Flavonoid OMTs are regioselective enzymes with wide substrate specificity [23]. Thus, only one enzyme could be involved in the methylation process. In addition, it is unlikely that several genes encoding OMTs were selected concurrently during the selection programs. More studies are required to gain more insights into the biochemical pathway of these *O*-methyl *C*-glycosylflavonoids. Comparative analysis of transcriptome of winter and spring flax should help to identify the missing steps of this pathway.

The link between the ability to produce and accumulate methylated *C*-glycosylated flavonoids and the cold tolerance of winter flax varieties still has to be demonstrated. However, work done in winter wheat has shown that cold stress induces the accumulation of *C*-glycosylflavonoids in the leaves after a cold treatment [24]. Other studies carried out on wheat have shown that this accumulation was particularly important for *O*-methylated flavonoids and that it was associated with a high total activity of OMTs in winter wheat leaves during cold acclimation [25]. All these data suggest that the presence of swertisin and swertiajaponin in winter flax could therefore contribute to cold tolerance in this plant cultivated in temperate climates. Further work must however be done to consolidate this hypothesis.

## 4. Materials and Methods

### 4.1. Experimental Design for Characterization of Phenolic Compounds

#### 4.1.1. Plant Materials

For purification of phenolic compounds, the cultivar of winter flax (Oleane) was used. Seeds come from Terre de Lin (France). Plants were cultivated in a greenhouse with a hemeroperiod of 16 h at 21 °C and a light intensity of 200 µmol·m^−2^·s^−1^. After 4 weeks, there were immersed in liquid nitrogen and then freeze-dried.

#### 4.1.2. Extraction

Leaves (50 g of dry weight) were ground to a fine powder. The powder was extracted at 70 °C for 20 min with 500 mL of methanol. Then 250 mL of chloroform were added to the mixture and the resulting solution was incubated for 10 min at 70 °C. Finally, 500 mL of H_2_O were added for phase separation. The hydroalcoholic fraction was collected and dried by evaporation to eliminate MeOH. The methanol free sample was then deposited on 100 g of an Amberlite^®^ XAD16N resin. A first elution was carried out with water (1 L) and a second with ethanol (500 mL). The ethanolic fraction was recovered and then dried by evaporation. This gave rise to approximately 1 g of dry residue.

#### 4.1.3. Purification by HPLC-UV

The dry residue (10 mg·mL^−1^) was injected on a LC-8A system (Shimadzu, Marne-la-Vallée, France) equipped with an Shimadzu SIL-10AE autosampler. The separation was performed on a Supelcosil™ABZ + Plus (5 μm × 25 cm × 21.2 mm) column (Sigma, St. Louis, MO, USA) at a flow rate of 17 mL·min^−1^. A gradient of H_2_O added with acetic acid (0.05%) (solvent A) and methanol (solvent B) was used for elution. Relative concentrations of solvents during the run were as follow: start at 100% solvent A; 30 min gradient to 50% solvent B; 10 min of isocratic 50% solvent B; 10 min gradient to 90% solvent B; 5 min gradient to 100% solvent A; 10 min of isocratic re-equilibration at 100% A. The fractions were collected with a FRC-10A fraction collector (Shimadzu). Detection was performed at 280 nm. For each fraction a second purification was performed to improve the purity. The collected fractions were diluted 10 times (MeOH/H_2_O) before LC-MS analyzes. For NMR analyzes, the collected fractions were dried by evaporation and then dissolved in MeOD–D_2_O (1:1).

#### 4.1.4. LC-MS Analysis of Fractions

UPLC-MS analyzes was carried on an ACQUITY UPLC I-Class system coupled to a Vion IMS QTof (Ion Mobility Quadrupole Time-of-flight) hybrid mass spectrometer, equipped with an electrospray ionization (ESI) source (Waters, Manchester, UK). One μL of each sample was injected and the chromatographic separation was performed on a Kinetex Biphenyl (100 × 2.1 mm, 1.7 μm) column (Phenomenex, Torrance, CA, USA), maintained at 55 °C. The mobile phase flow was set to 0.55 mL/min and a gradient elution going from water with formic acid 0.1% (A) to methanol with formic acid 0.1% (B) was programmed as follows (A:B): 80:20 (t = 0 min), 80:20 (t = 0.5 min), 40:60 (t = 5 min), 10:90 (t = 6 min), 10:90 (t = 7 min), 80:20 (t = 7.5min), 80:20 (t = 10 min). The ESI source has been set to a 2.5 kV capillary voltage in negative ionization mode with a 20 V sampling cone voltage. The source and desolvation temperatures were set at 120 °C and 450 °C, respectively. Nitrogen was used as desolvation and cone gas at flow rates of 800 and 50 L/h, respectively. For accurate mass measurements, a lock mass correction was applied using the [M-H]^-^ ion at m/z 554.2615 of a Leu-enkephalin solution (100 pg/μL in H_2_O/CH_3_CN (50/50 *v/v*) with formic acid 0.1%). The TOF was operated in the sensitivity mode, providing an average resolving power 50,000 (FWHM). The MS spectra were recorded in the profile mode over the 50–2000 m/z mass range with a scan time set to 0.2 s. For MS/MS experiments, argon was used as collision gas and the collision energy value (see Supplementary Materials 2) was optimized to reach a relative intensity between 10–20% for each selected precursor ion. Data acquisition was performed with UNIFI software (V1.9.2, Waters).

#### 4.1.5. NMR Analysis of Fractions

All the fractions were transferred into 5-mm NMR tubes. NMR spectra were acquired at 300 K on an Avance III 600 spectrometer (Bruker Biospin, Wissembourg, France) operating at 600.17 MHz for ^1^H, using a z-gradient inverse probe head (TXI 5 mm tube). The TOPSPIN v3.2 (Bruker) software was used. The 1D classic proton with 90° flip was performed. Each spectrum consisted of 64 scans of 128 K data points with spectral width of 8417 Hz. 2D ^1^H JRES NMR spectra were acquired using 8 transients per 64 increments that were collected into 64 K data points, using spectral widths of 8417 Hz along the direct dimension and 50 Hz along the indirect dimension, using excitation sculpting for the water suppression. The 1D DEPTQ spectra were acquired using 6 K scans of 128 K data points, using spectral widths of 37,878 Hz. The 2D COSY spectra were acquired using 8 scans per 256 increments that were collected into 4 K data points, using spectral widths of 8417 Hz in both dimensions. The 2D TOCSY spectra were acquired with 8 scans per 256 increments that were collected into 4 K data points, with spectral widths of 8403 Hz in both dimensions. A mixing time of 0.1 s were employed. The 2D HSQC spectra were acquired using 32 scans per 256 increments that were collected into 2 K data points, using spectral widths of 8417.5 Hz in F2 and 26,412 Hz in F1. The 2D HMBC spectra have been acquired using 32 scans per 512 increments that were collected into 4 K data points, using spectral widths of 8417.5 Hz in F2 and 37,732 Hz in F1. 

#### 4.1.6. Chemical Products

Standard compounds for identification by UPLC-MS were provided by Sigma (St. Louis, MO, USA) for coniferin, isoorientin, isovitexin, orientin, schaftoside, vicenin-2, vitexin; by Ambinter (Orléans, France) for carlinoside, LMG, vicenin-1; by Chemfaces (Wuhan, Hubei, China) for PMG, swertiajaponin, swertisin; by Carbosynth (Compton, Berkshire, UK) for lucenin-2.

### 4.2. Experimental Design for Phenolic Compounds Profiling

#### 4.2.1. Plant Materials

Three winter flax varieties (Blizzard, Orival, Volga) and three spring flax varieties (Altess, Aries, Marquise) were grown hydroponically as described in [26]. Seeds of the variety Blizzard, Altess and Marquise were provided by Linea (France). Seeds of the varieties Aries, Volga and Orival come from Limagrain (France), Terre de Lin (France) and Laboulet (France), respectively. Plants were grown in a greenhouse which parameters were set as follow: 21 °C with a light intensity of 200 µmol·m^−2^·s^−1^ and a hemeroperiod of 16 h. After 2 weeks, leaves were collected, fixed in liquid nitrogen and freeze-dried. For profiling of phenolic compounds, extraction has been performed as described in [27]. For each variety, 10 plants were independently cultivated, extracted and analyzed (*n* = 10).

#### 4.2.2. LC-MS Analysis

Metabolite profiling was carried on polar extracts diluted ten times with water/methanol (1:1). UPLC-MS analyzes were performed as described in Section 4.1.4. All information about identification and quantification are indicated in the Supplementary Materials 2.

#### 4.2.3. Data Analysis

Principal component analysis (PCA) was performed with the R software (v. 3.6.1, company Foundation for Statistical Computing, Vienna, Austria) based on an univariate scaling method. The non-parametric Kruskall-Wallis tests were performed with the R software and the multiple comparison following the Kruskall-Wallis tests were performed using the PMCMR (v. 4.3) and multcompView (v. 0.1-7) packages to test significant differences (*p*-value < 0.05) between all the varieties.

## Supplementary Materials

The following are available online. Supplementary Materials 1: Structure and NMR table of swertisin, Supplementary Materials 2: LC-MS informations, Supplementary Materials 3: Structure and NMR table of swertiajaponin, Supplementary Materials 4: Structure and NMR table of triticuside A, Supplementary Materials 5: Structure and NMR table of HHMPG.
