# Supplementary material for: Phenolic Profiling of Flax Highlights Contrasting Patterns in Winter and Spring Varieties
Source: Molecules. 2019 Nov 26;24(23):4303. doi: 10.3390/molecules24234303 (PMC6930658; doi:10.3390/molecules24234303)

**Supplementary materials 1.** NMR table summarizing for swertisin the assignment of $^{13}C$ and $^{1}H$ chemical shifts (δ in ppm) and $^{1}H$–$^{1}H$ coupling constants (*J* in Hz) obtained from 1D and 2D NMR spectra recorded in $D_2O$ / $CD_3OD$ (50/50 *v*/*v*) at 300 K.

| No | $^{13}C$ | $^{1}H$ |
|---|---|---|
| 1 | - | - |
| 2 | 165.1 | - |
| 3 | 102.9 | 6.69 |
| 4 | 182.9 | - |
| 5 | 165.1 | - |
| 6 | 109.0 | - |
| 7 | 165.1 | - |
| 8 | 89.9 | 6.77 |
| 9 | 158.0 | - |
| 10 | 104.8 | - |
| 1' | 121.6 | - |
| 2' | 128.0 | 7.91 (d, *J* = 8.8) |
| 3' | 115.8 | 6.95 (d, *J* = 8.8) |
| 4' | 161.6 | - |
| 5' | 115.8 | 6.95 (d, *J* = 8.8) |
| 6' | 128.0 | 7.91 (d, *J* = 8.8) |
| 1'' | 73.0 | 4.90 (d, *J* = 10.3 ) |
| 2'' | 70.9 | 4.23 (t, *J* = 9.5 ) |
| 3'' | 79.0 | 3.44 (m) |
| 4'' | 70.9 | 3.39 (m) |
| 5'' | 81.2 | 3.39 (m) |
| 6'' | 61.9 | 3.68 (m) |
| | | 3.88 (br d, *J* = 12.4) |
| 7'-OMe | 55.3 | 3.96 (s) |

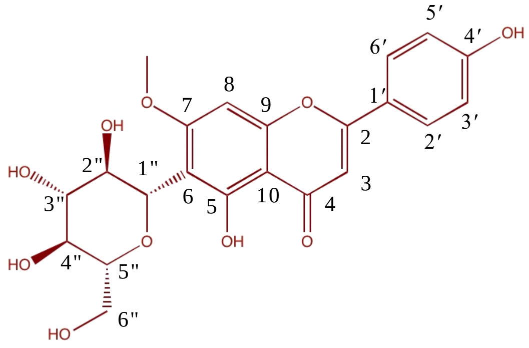

Supplement: Supplementary file 1 [file molecules-24-04303-s001.zip › molecules-630107-SM-final/Supplementary materials1.pdf]
