# Supplementary material for: Phenolic Profiling of Flax Highlights Contrasting Patterns in Winter and Spring Varieties
Source: Molecules. 2019 Nov 26;24(23):4303. doi: 10.3390/molecules24234303 (PMC6930658; doi:10.3390/molecules24234303)

Supplementary materials 3. NMR table summarizing for swertiajaponin the assignment of ^{13}C and ^{1}H chemical shifts (δ in ppm) and ^{1}H–^{1}H coupling constants (*J* in Hz) obtained from 1D and 2D NMR spectra recorded in D_2O/CD_3OD (50/50 *v*/*v*) at 300 K.

No	^{13}C	^{1}H
1	-	-
2	165.0	-
3	102.6	6.62 (s)
4	183.1	-
5	164.5	-
6	109.0	-
7	165.0	-
8	89.5	6.74 (s)
9	158.0	-
10	104.5	-
1'	122.0	-
2'	112.6	7.41 (d, *J* = 2.1)
3'	145.7	-
4'	149.8	-
5'	115.0	6.92 (d, *J* = 8.4)
6'	118.6	7.44 (dd, *J* = 2.1/8.4)
1''	72.7	4.89 (d, *J* = 10.0)
2''	70.4	4.23 (t, *J* = 9.3)
3''	78.8	3.43 (m)
4''	70.4	3.28 (m)
5''	80.9	3.38 (m)
6''	61.6	3.67 (m)
		3.88 (br d, *J* = 12.0)
7'-OMe	55.0	3.95 (s)

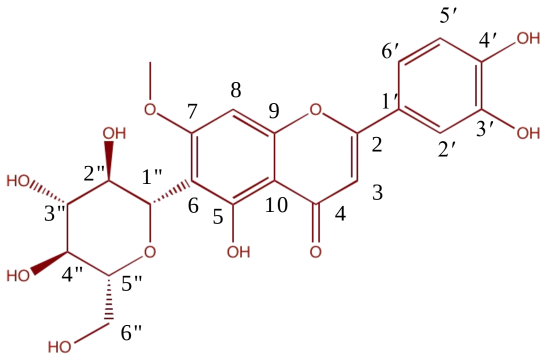

Supplement: Supplementary file 1 [file molecules-24-04303-s001.zip › molecules-630107-SM-final/Supplementary materials3.pdf]
