# Supplementary material for: Phenolic Profiling of Flax Highlights Contrasting Patterns in Winter and Spring Varieties
Source: Molecules. 2019 Nov 26;24(23):4303. doi: 10.3390/molecules24234303 (PMC6930658; doi:10.3390/molecules24234303)

Supplementary materials 4. NMR table summarizing for triticuside A the assignment of ^{13}C and ^{1}H chemical shifts (δ in ppm) and ^{1}H–^{1}H coupling constants (*J* in Hz) obtained from 1D and 2D NMR spectra recorded in D_2O/CD_3OD (50/50 v/*v*) at 300 K.

No	^{13}C	^{1}H
1	-	-
2	165.4	-
3	102.5	6.67 (s)
4	182.9	-
5	157.9	-
6	109.2	-
7	161.5	-
8	103.1	-
9	156.3	-
10	104.1	-
1'	122.3	-
2'	128.8	8.07 (d, *J* = 8.9)
3'	115.8	6.98 (d, *J* = 8.9)
4'	161.5	-
5'	115.8	6.98 (d, *J* = 8.9)
6'	128.8	8.07 (d, *J* = 8.9)
1''	124.7	-
2''	105.7	6.87 (s)
3''	148.0	-
4''	138.4	-
5''	148.0	-
6''	105.7	6.87 (s)
7''	145.9	7.47 (d, *J* = 15.9)
8''	114.3	6.21 (d, *J* = 15.9)
9''	167.1	-
1'''	75.5	4.81 (d, *J* = 9.8)
2'''	70.7	3.76 (m*)
3'''	73.9	3.50 (m*)
4'''	68.8	3.90 (m*)
5'''	81.7	3.53 (m*)
6'''	61.5	3.83 (m*)
		3.99 (m*)
1''''	71.4	5.24 (d, *J* = 10.1)
2''''	72.8	5.71 (dd, *J* = 9.1/10.1)
3''''	76.6	3.82 (m*)
4''''	70.6	3.77 (m*)
5''''	70.8	3.70 (m*)
		4.02 (m*)
3''-OMe	55.3	3.87 (s)
5''-OMe	55.3	3.87 (s)

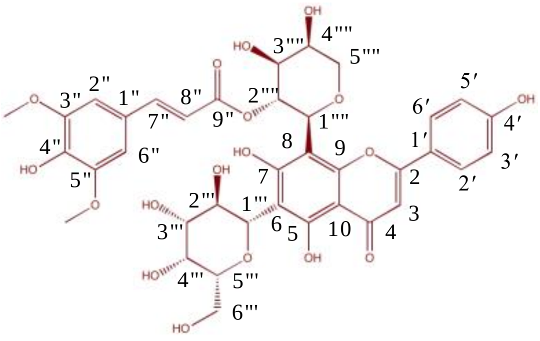

* The multiplicity of the signal is difficult to observe due to overlapping with other signals and is described as multiplet (m).

MS/MS spectrum performed on the [M-H]$^{-}$ ion at *m/z* 769.20 (Collision Energy 23eV) corresponding to the chromatographic peak at R_t 4.91 min.

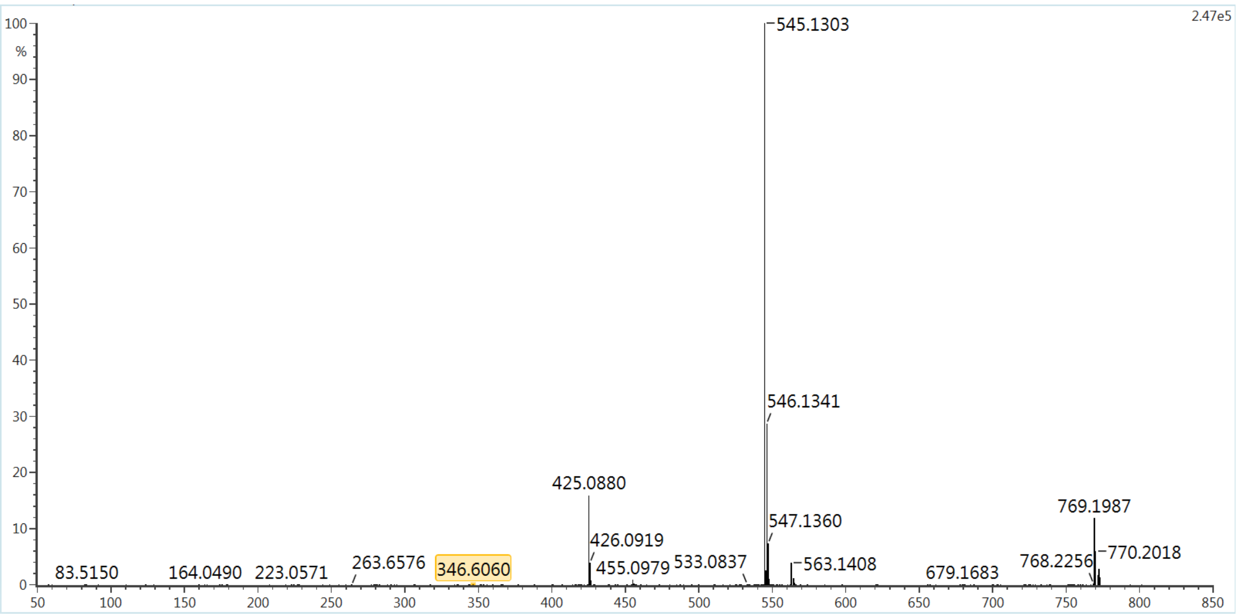

MS/MS spectrum performed on the [M-H]$^{-}$ ion at *m/z* 769.20 (Collision Energy 23eV) corresponding to the chromatographic peak at R_t 4.82 min.

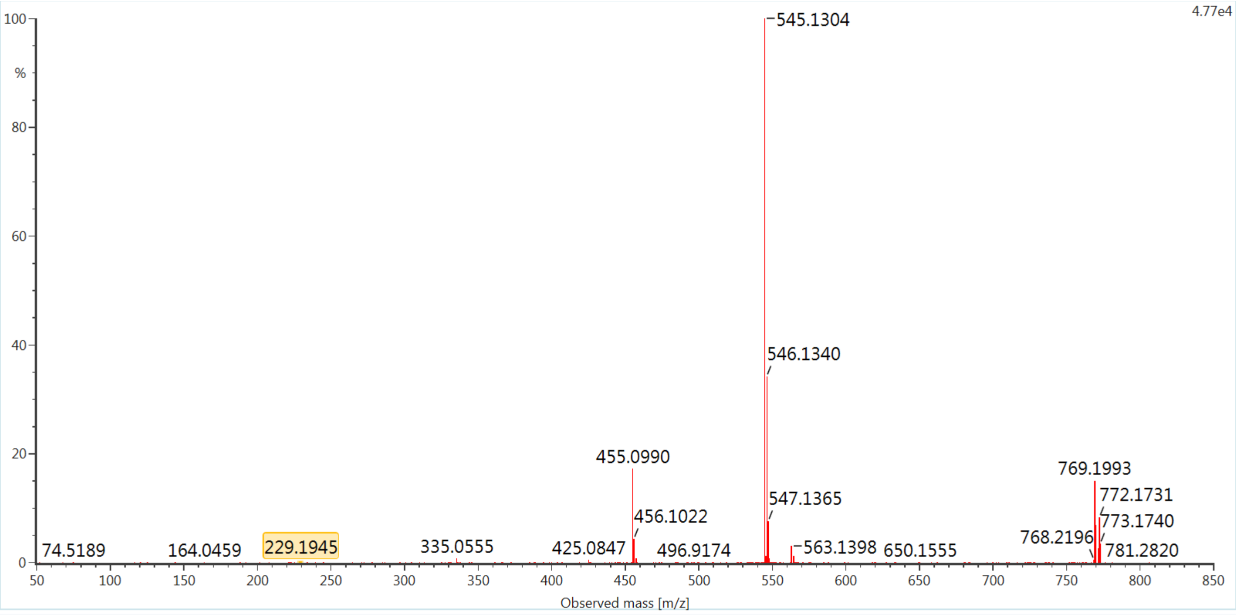

Hypothetic fragmentation pathway based on the fragment ions obtained by MS/HRMS from triticuside A deprotonated molecule

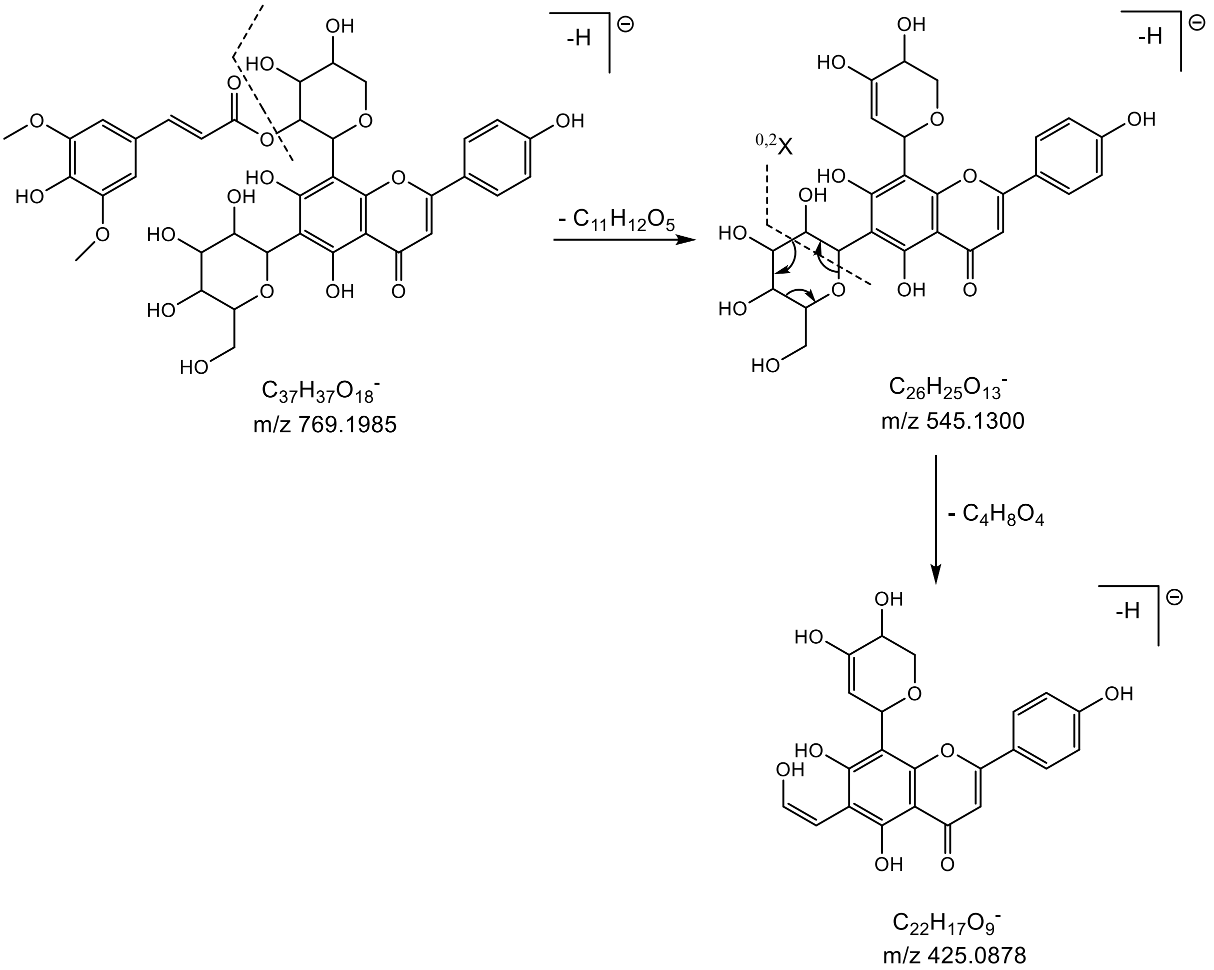

Supplement: Supplementary file 1 [file molecules-24-04303-s001.zip › molecules-630107-SM-final/Supplementary materials4.pdf]
