# Supplementary material for: Phenolic Profiling of Flax Highlights Contrasting Patterns in Winter and Spring Varieties
Source: Molecules. 2019 Nov 26;24(23):4303. doi: 10.3390/molecules24234303 (PMC6930658; doi:10.3390/molecules24234303)

Supplementary materials 5. NMR table summarizing for HHMPG the assignment of ^{13}C and ^{1}H chemical shifts (δ in ppm) and ^{1}H–^{1}H coupling constants (*J* in Hz) obtained from 1D and 2D NMR spectra recorded in D_2O/CD_3OD (50/50 *v*/*v*) at 300 K.

No	^{13}C	^{1}H
1	60.6	3.80 (dd, *J* = 4.7/12.2)
2	79.6	4.42 (m)
3	67.8	3.84 (m)
		4.11 (m)
1'	146.9	-
2'	150.9	-
3'	110.0	7.06 (d, *J* = 2.1)
4'	131.9	-
5'	119.3	6.94 (dd, *J* = 2.1/8.2)
6'	117.5	7.07 (d, *J* = 8.2)
7'	130.0	6.56 (br d, *J* = 15.9)
8'	127.3	6.28 (dt, *J* = 5.7/15.9)
9'	62.3	4.21 (dd, *J* = 1.5/5.7)
1''	103.3	4.33 (d, *J* = 7.8)
2''	73.7	3.20 (dd, *J* = 7.8/9.8)
3''	76.5	3.35 (m)
4''	70.8	3.30 (m)
5''	76.6	3.29 (m)
6''	61.5	3.66 (m)
		3.87 (m)
2'-OMe	55.1	3.86 (s)

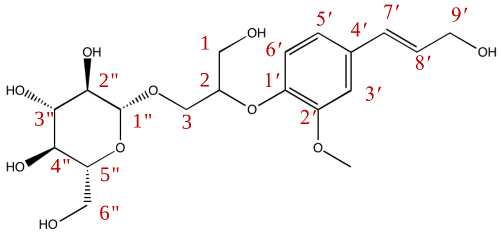

Supplement: Supplementary file 1 [file molecules-24-04303-s001.zip › molecules-630107-SM-final/Supplementary materials5.pdf]
